# *MedComm* announcement

**DOI:** 10.1002/mco2.3

**Published:** 2020-04-25

**Authors:** James Henderson Naismith, Yu‐quan Wei

**Affiliations:** ^1^ Rosalind Franklin Institute the University of Oxford Oxfordshire UK; ^2^ State Key Laboratory of Biotherapy and Clinical Cancer Center West China Hospital Sichuan University Chengdu China

Molecular medicine is emerged with the progression of life science, representing the third medicine revolution after clinical anatomy and experimental medicine in the last centuries. As a multidisciplinary field, molecular medicine integrates physics, chemistry, biology, and medical technology, which aims at deciphering the structural, molecular, and cellular mechanisms that drive pathogenic process and at developing molecular tools and interventions for the diagnosis, prevention, and treatment of human diseases. A milestone in molecular medicine is the launch of Human Genome Project (HGT) in 1990, promoting the advance of diverse subdisciplines including epigenetics, genomics, transcriptomics, proteomics, and metabolomics. As a result, many new pathogenic factors are uncovered in recent years, such as gene mutations in inherited disease, posttranslational modification in aging, and noncoding RNAs in cancer. Assisting by translational medicine, the cutting‐edge academic theories and technologies of molecular medicine have been developed into products that improve human health, such as nivolumab (Opdivo; Bristol‐Myers Squibb, New York, NY, USA) for the treatment of nonsmall cell lung cancer, Adalimumab (Humira; AbbVie, Chicago, IL, USA) for the treatment of rheumatoid arthritis, and artificial intelligence‐driven platform (Exscientia, Oxford, UK) for drug discovery.

With the progress of technologies and the continuous efforts of researchers, there is an advance in our understanding of molecular and translational medicine. However, many challenges are still to be faced. For example, the regulatory network consisting of proteins and nucleic acids is far from understood. Existing targeted therapies for diseases are unable to meet clinical needs. Precise diagnostic technologies, especially for early disease diagnosis, are also inadequate. Growing publications have suggested that this field is a rapidly expanding realm of research. Based on a PubMed Advanced Search (All Fields: “molecular medicine”), the total number of papers published in 1985 was 47 and increased to 12 260 in 2002, and further reached to 60 452 in 2018. However, high‐impact journals in this field are limited, as a result, the published articles are vastly decentralized. Furthermore, governments and foundations around the world are increasingly providing financial support to the research in molecular medicine, including molecular diagnosis, molecular pathology, signal transduction, targeted intervention, drug discovery and delivery, and other clinical techniques for managing human diseases. It is expected that more and more important discoveries will be achieved in the near future. However, the limited capacity of existing journals cannot ensure the timely publication of relevant works with an increasing rate. Thus, we are launching *MedComm* to meet the current and future demand, providing a home for this field.

*MedComm* is a peer‐reviewed, online open access journal that publishes the pioneer works of medicine on the basis of novelty, timeliness, and significance on human health. Notably, *MedComm* is a multidisciplinary journal, covering disciplines including but not limited to clinical medicine, molecular biology, cell biology, pharmacology, medicinal chemistry, and pharmacy. Areas of interest contain basic researches that advance the understanding of pathogenesis, such as clinical epigenetics, genomics, proteomics, metabolomics, noncoding RNA, microenvironment, and cellular signal transduction. Researches that improve the diagnosis and treatment of human diseases, such as early disease diagnosis, liquid biopsy, high‐definition imaging, gene therapy, cell therapy, immunotherapy, chemical genomics, drug discovery, artificial intelligence in medicine, regenerative medicine, medical nanotechnology, drug delivery, and gene editing, are also interested by *MedComm*. All the major human diseases, including cardiovascular diseases, cancer, neurodegenerative disease, autoimmune disorders, and other pathologies, are within the scope of *MedComm*. It has a wide range of authors and readership, including researchers engaged in basic and applied research related to molecular medicine and translational medicine, clinicians concerned with pathogenic mechanism, as well as students in medicine, life science, and drug discovery.

We aim to establish a leading forum for medical research. Research articles section, letters section, and reviews section are open for authors to submit their pioneer works, viewpoints, and summaries in the frontiers of medicine. We will also invite experts to publish cutting‐edge reviews in this field. In addition, *MedComm* will aperiodically provide news, views, and highlights to comment on the latest medical progress, which timely address controversial topics in medicine.

The editorial team of *MedComm* is composed of high‐impact scientists from all over the world, and welcomes researchers to submit valuable works to *MedComm*. We promise fast review followed by fair and prompt decisions. Once a manuscript is accepted, our editorial staffs will provide high‐quality support to ensure your article is the best that it can be.

With advances in high‐throughput sequencing and high‐resolution analysis technologies, novel pathogenic mechanisms, biomarkers, and targets are continuously revealed, followed by the emergence of new techniques for the diagnosis, prevention, and treatment of human disease. *MedComm* provides a platform for students, scientists, and clinicians interested in clinical, basic, and translational medicine to share their discoveries and viewpoints in this field. We are committed to operating the *MedComm* innovative and timely, aiming to offer valuable and helpful information to you and the community.

